# Anorectal Dysfunction in Multiple Sclerosis: A Systematic Review

**DOI:** 10.5402/2012/376023

**Published:** 2012-07-29

**Authors:** Sanober Nusrat, Elsie Gulick, David Levinthal, Klaus Bielefeldt

**Affiliations:** ^1^Division of Gastroenterology, Hepatology and Nutrition, Department of Medicine, University of Pittsburgh Medical Center, Pittsburgh, PA 15217, USA; ^2^Rutgers College of Nursing, The State University of New Jersey, 180 University Avenue, Newark, NJ 07102, USA

## Abstract

Constipation and fecal incontinence are common in patients with neuromuscular diseases. Despite their high prevalence and potential impact on overall quality of life, few studies have addressed anorectal dysfunction in patients with multiple sclerosis (MS). The goal of this paper is to define the prevalence, pathophysiology, impact, and potential treatment of constipation and incontinence in MS patients. *Methods*. The PubMed database was searched for English language publications between January 1973 and December 2011. Articles were reviewed to assess the definition of the study population, duration, type and severity of MS, sex distribution, prevalence, impact, results of physiologic testing, and treatments. *Results*. The reported prevalence of constipation and fecal incontinence ranged around 40%. Anorectal dysfunction significantly affected patients with nearly 1 in 6 patients limiting social activities or even quitting work due to symptoms. Caregivers listed toileting as a common and significant burden. The only randomized controlled trial showed a marginal improvement of constipation with abdominal massage. All other reports lacked control interventions and only demonstrated improvement in individuals with milder symptoms. *Conclusion*. Anorectal dysfunction is a common manifestation in MS that significantly affects quality of life. Therapies are at best moderately effective and often cumbersome, highlighting the need for simple and more helpful interventions.

## 1. Introduction

The diagnostic and therapeutic approach to multiple sclerosis (MS) has changed with the introduction of improved neuroimaging technology and disease-modifying medications. A decrease in the frequency and severity of relapses with a positive impact on overall functional abilities led to calls for earlier treatment initiation in patients with relapsing remitting MS [1]. The need to identify potentially eligible patients shifted the emphasis from clinical relapse to more sensitive biomarkers of disease activity, with magnetic resonance imaging being the most widely accepted and used surrogate marker [2]. Consistent with the results of clinical trials, large cohort studies suggest a slower progression of disability [3, 4]. However, many patients continue experiencing worsening physical and cognitive problems [5]. While the assessment of disability includes many of the functional systems typically affected by multiple sclerosis, the emphasis is on ambulation or other physical functions [6, 7]. Visceral functions are also quite often impaired and can have a significant impact on the quality of life of affected patients. Most of our understanding related to the visceral manifestations of MS focuses on the abnormalities of micturition. Yet, clinical practice and limited data suggest that the gastrointestinal tract is often involved. Treatment options are limited and have not been evaluated in larger and appropriately designed trials. Most studies focus on constipation or incontinence, which are often found in patients with micturition problems. The goal of our systematic review is to define the prevalence of constipation and incontinence in MS patients, to determine the underlying pathophysiology, and to assess the outcome of different treatment strategies. Another dimension that is often overlooked is the indirect impact of progressive loss of function that comes with the need for ongoing care, which typically falls to the MS patient's informal caregiver, mostly the spouse or partner [8, 9]. This need for ongoing assistance has become increasingly important as most persons with chronic disabling diseases want to be cared for in their familiar surroundings and as health care providers recognize the psychological and social benefits of home-caring. On the other side, the growing economic constraints in the health care system limit hospital admissions and shorten inpatient stays. Our paper thus went beyond the traditional approach seeking to understand the manifestations of an illness and also included information about the care giver burden of those with MS. Based on these data, we will propose potential treatment options and areas for further study. 

## 2. Methods

Using the PubMed database, we performed a systematic analysis of clinical trials and case series published in English between 1973 and 2011. The keywords used to search the literature were “MS and constipation”, “MS and fecal incontinence,” “MS and anorectal dysfunction,” “MS and anal sphincter,” and “MS and care giver.” In addition, references of selected articles were reviewed to identify additional potentially relevant studies. Studies were excluded if they focused on pediatric patients, were isolated case reports, editorials, letters to the editor, reviews, or did not provide detailed information on gastrointestinal symptoms. 

We grouped the search into the following three domains: MS and constipation, MS and fecal incontinence, and anorectal dysfunction in MS and care giver burden. Articles were reviewed to assess the definition of the study population, duration, type and severity of MS, sex distribution, prevalence, type and severity of gastrointestinal manifestations with a focus on constipation and fecal incontinence, and results of physiologic testing. For reports on treatment of gastrointestinal problems, we also abstracted trial design, definition of endpoints, duration of treatment and followup as well as response rates. To eliminate potential bias due to double publications, all data were entered into a computer-based system and searched for overlap in contributing authors. The studies were re-reviewed to determine the timeframe of patient recruitment. In cases of overlap, only one of the multiple publications was used. If the patient recruitment only partially overlapped, we selected the article with largest patient sample. 

## 3. Results

**Figure 1:**
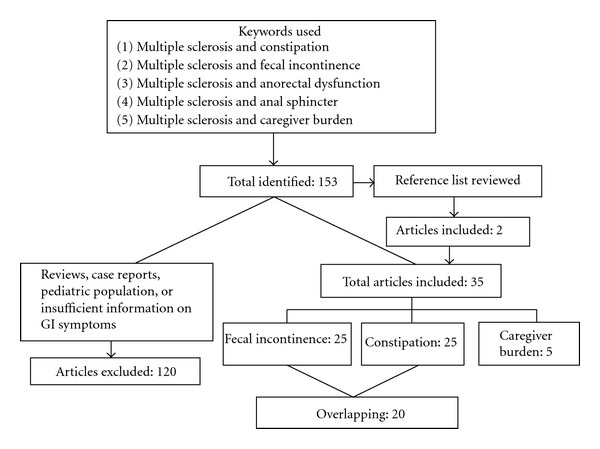
Strategy and results of the PubMed search.

The electronic search identified a total of 153 publications of which 33 articles met our inclusion criteria. An additional 2 studies were included based on review of references in these publications. Of the 35 articles, 25 provided information on constipation, 25 addressed FI, with a significant overlap as 20 describing the prevalence of both manifestation of anorectal dysfunction. No article specifically addressed caregiver burden and anorectal dysfunction in MS patients. Widening the criteria, we identified 9 articles that addressed the burden of fecal incontinence on partners, spouses, and caregivers. However, these publications did not include MS patients. Shifting to the terms *multiple sclerosis *and *caregiver burden*, we found 45 citations with 5 addressing issues related to defecation disorders or incontinence (Figure 1).

### 3.1. Prevalence of Fecal Incontinence and Constipation in MS

The definition of constipation was variable, with most studies relying on published consensus criteria [10–18]. A few studies combined physiologic markers (i.e., whole gut transit time) with reported defecation frequency [19], relied on patient self-assessment [20, 21] or a validated scoring system [22], or based the definition of constipation on discomfort during defecation, a feeling of incomplete emptying after defecation [23] and/or the need for digital manipulation, laxative use or failure or prior treatment for constipation [24, 25]. Similarly, the definition of fecal incontinence also varied and was primarily based on the frequency of incontinence episodes, typically described as the involuntary emission of flatus or stool at least once [10, 15, 18, 23]or with repeated episodes during the course of their disease [12, 18, 22]. Six additional studies were more restrictive and assessed incontinence episodes within the last 1–3 months [13, 16, 17, 21, 26, 27]. Finally, four studies relied on standardized questionnaires to define fecal incontinence [11, 20, 25, 28]. One large study used nursing home admission notes to identify the prevalence of fecal incontinence in a large sample of MS patients [29]. 

Combining all data, 1,975 MS patients and 217 controls were included in studies describing the prevalence of constipation. Most studies were small with sample sizes <100. Five studies recruited both MS patients and control groups [12, 15, 17, 18, 30]. One additional article described hospitalizations of MS patients and reported the number attributed to constipation and/or its complications [31]. Publications that focused on fecal incontinence include 16,072 MS patients and 441,017 controls. As was the case for studies on constipation, most studies included <100 patients. However, one large study examined the prevalence of incontinence in nursing home residents based on a large registry of patients with and without MS [29], skewing data due to the higher prevalence of significant functional impairment. Control groups were included in 9 studies [12, 15, 17–19, 26, 27, 29, 32]. Consistent with the epidemiology of MS, patient samples showed a female predominance of about 2-3:1, with most patients being classified as having moderate disease severity based on standardized assessment scales or self-reporting. The largest patient sample included focused on nursing home residents, in whom more than 50% depended on assistance in activities of daily living [29]. Data on disease type were limited and relied on patient report in studies with larger sample size. The majority of patients had a relapsing remitting form of MS, followed by a secondary progressive type with typically less than 15% having primary progressive MS. 

**Figure 2:**
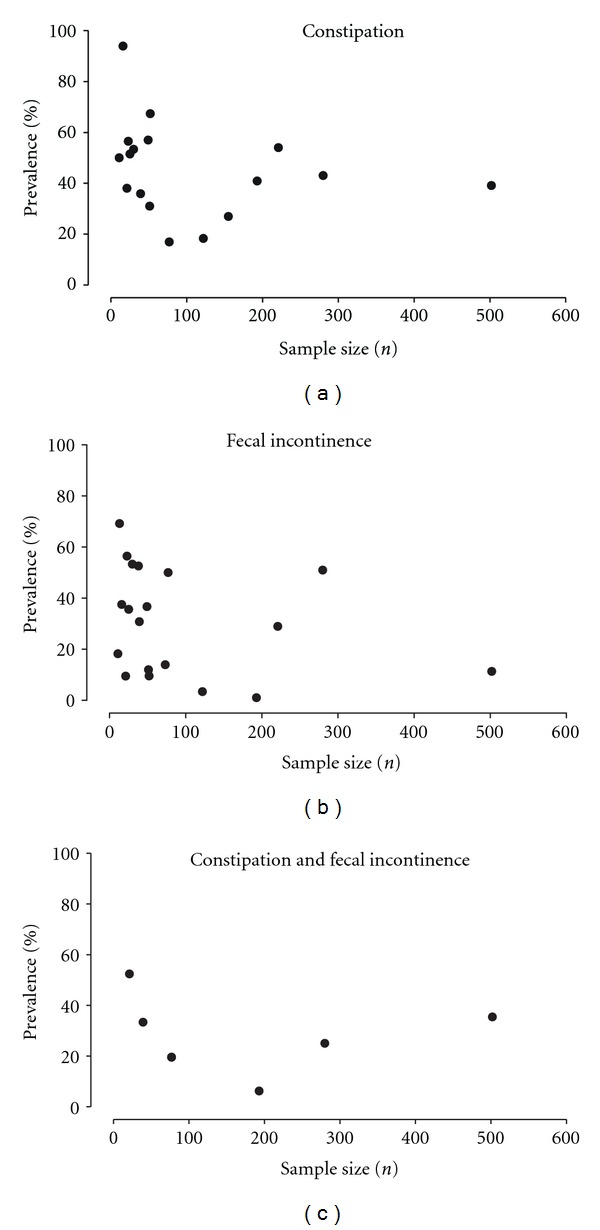
The scatter plots show the study populations and prevalence of (a) constipation, (b) fecal incontinence, and (c) coexisting constipation and fecal incontinence in MS patients.

The prevalence of constipation ranged from 17–94% (Figure 2(a)) with studies including at least 100 patients narrowing the range to 18–43% of MS patients. The wide range is partially due to the different recruitment mechanisms, differences in the duration and severity of illness and the definition of constipation. Data on fecal incontinence similarly showed a wide spread from 1–69% (Figure 2(b)). The lowest prevalence was seen in a study with a more restrictive definition of fecal incontinence, requiring multiple episodes within the month preceding study inclusion [16]. Except for this study, studies with sample sizes of at least 100 patients reported data between 3.4 and 51% [10, 17, 20, 29] with the lowest number possibly being skewed due to limited assessment of anorectal dysfunction in a study that focused on problems with micturition [33]. A mixed form of anorectal dysfunction was reported for total patient sample of 1,292 MS patients with a reported prevalence between 6 and 52% (Figure 2(c)) [10, 13, 16, 17, 22, 23, 25]. Nine studies examined the correlation between the overall functional impairment and bowel symptoms, with five reporting a higher prevalence of anorectal dysfunction with progressive worsening of functional status [15–17, 26, 33]. These findings correlated with the higher likelihood of defecation problems in patients with progressive forms of MS and longer disease duration, both of which relate to a decline in functional status [15, 17].

### 3.2. Mechanisms of Anorectal Dysfunction

Most studies employed pressure sensing devices within the anal canal to assess the function of sphincteric muscles. As shown in Table 1, results showed a normal or slightly decreased resting and a significantly decreased squeeze pressure in most studies focusing on patients with constipation. Similar findings were reported in patients suffering from fecal incontinence with an even higher incidence of impaired function of the external anal sphincter, confounded by sex and prior obstetric trauma, a typical finding that has been reported in disease controls (Table 1). The resting pressure is largely determined by the function of the intrinsically controlled internal anal sphincter, while the increase during voluntary sphincter squeeze reflects the volitionally controlled function of the external sphincter [34]. Considering the clinical manifestations of MS, several studies also investigated sensory thresholds in response to rectal distension with only two studies showing impaired sensation in MS patients (Table 1). A single study provided additional details about defecation dynamics, describing a limited generation of intra-abdominal pressure during straining in the majority of constipation MS patients [11]. A second study relied on different approaches and performed defecography in MS patients. The limited effacement of the puborectalis muscle suggested impaired defecation dynamics due to limited relaxation of sphincteric structures [24]. Only one study addressed whole gut transit time, reporting normal findings in MS patients with fecal incontinence and a slow transit in constipated patients [19]. In a small study, a slow rate of water infusion into the colorectum triggered a steeper pressure rise with apparent reflex contractions in MS patients with fecal incontinence compared to controls [12]. As results are confounded by filling of the more proximal colon, it remains unclear whether these observations truly demonstrate hyperreflexia.

### 3.3. Treatment of Anorectal Dysfunction

One survey addressed the frequency of use and the subjectively perceived utility of treatments patients employed to treat anorectal disorders [30]. Consistent with the high prevalence of constipation, more than half of the respondents used laxatives, suppositories, and/or enemas, with about 60% rating them to be at least moderately effective. About one-fifth of the patients required digital stimulation or even manual help with rectal emptying; as was true for the different medical interventions, slightly more than 60% of the respondents considered these manipulations to be moderate to very helpful. One-fourth of the participants employed abdominal massage; in contrast to the other approaches, only one-third viewed this strategy as at least moderately helpful. 

Studies of treatment for anorectal disorders in MS patients are largely restricted to small case series. Most reports showed a similar subjective recall of efficacy across a wide range of different therapeutic approaches (Table 2). A single randomized controlled trial has been conducted that compared the effects of abdominal massage with educational and lifestyle measures only [14]. In this study, while composite scores of bowel dysfunction improved more with the active intervention for both time points assessed during the study, bowel frequency only increased at 4 weeks and was not reported to be different from control interventions at 8 weeks. Most of the uncontrolled trials employed gut-focused behavioral therapy augmented by biofeedback. Approaches were not standardized, with the design of biofeedback sessions, their total number and time between sessions varying between less than three and more than ten training encounters. In addition, trials included educational efforts, such as giving toileting advice or optimizing the use of laxatives and suppositories, limiting our ability to correlate the outcome with a single intervention. As shown in Table 2, response rates were around 40% independent of the strategy chosen and/or the duration of therapy. Improvement was more likely in patients with lower disability scores and less severe symptoms. Retrospective analyses of transanal irrigation reported improved symptoms with similar response rates [25]. While potentially promising, the time requirements for transanal irrigation were a major concern for many patients who eventually discontinued the use of this intervention. Interestingly, discontinuation of therapy was also defined as response, which may potentially inflate the reported results. 

### 3.4. Impact of Anorectal Dysfunction in MS Patients

Few studies systematically addressed the impact of bowel dysfunction on the overall quality of life in MS patients. As anorectal problems often coincided with urogenital dysfunction, patients frequently report negative effects on their sex life and intimate relationships [28, 33]. One study specifically asked patients to identify domains that had been affected by their bowel dysfunction [30]. Nearly half of the respondents (47%) stated that they had adjusted their lives to accommodate their bowel regimen, with 15% spending more than 30 min each day to implement this bowel regimen. About one-fifth of the sample (19%) judged their social life as being significantly affected by their bowel problems, and 15% stated that these problems had forced them to stop working outside of their homes. 

### 3.5. Caregiver Burden and Anorectal Dysfunction

While none of the articles focused on anorectal dysfunction, they identified the primary caregivers, their involvement, and the subjectively perceived burden. About 60–70% of the caregivers are spouses of MS patients, spending anywhere from a few minutes to more than 3 hours a day to provide support in many activities of daily living [8, 38]. The amount of time required to care for MS patients increases with the progression of disability [8], a factor that correlates with the development of anorectal dysfunction [15, 17]. Studies detailing the kind of support needed listed toileting or perineal care in 28–48% [39, 40]. About 60% of MS patients and 25% of their caregivers listed help for continence problems as having a significant negative impact [41]. This is consistent with ratings provided in a survey of caregivers, who rated incontinence as one of the most distressing symptoms [42]. 

## 4. Discussion

Our systematic review of the published literature certainly shows that anorectal dysfunction is very common in MS patients. It also demonstrates the potentially significant impact on quality of life and functional status, as many patients regularly spent time and effort to implement some form of bowel regimen. The impact goes beyond the physical and emotional toll anorectal dysfunction takes on patients. Many caregivers spent significant effort to assist their spouses or partners in these daily tasks, adding to the burden and often contributing to ultimate decision to move patients into a nursing home [43]. This hidden disability thus contributes to the loss of professional productivity, social isolation and often constitutes a tremendous burden on intimate relationships. Despite the obvious relevance, few studies have explored treatment options, with only one single controlled, but underpowered investigation, ever having been completed in MS patients with anorectal dysfunction. 

As is true for many other manifestations of MS, anorectal dysfunction tends to develop and worsen as the disease progresses. Consistent with the expected patterns of a demyelinating disease of the central nervous system, physiologic investigations largely revealed impaired responses to the volitionally controlled sphincteric mechanisms, which certainly contribute to fecal incontinence. Based on radiographic investigations, the altered central control of pelvic floor muscles may result in spasticity and thus impair the ability to successfully evacuate rectal contents. Weakness and/or spasticity may also limit a patient's ability to initiate defecation by straining effectively. Beyond changes in neuromuscular function, some studies implicate altered visceral sensory mechanisms, which may lead to impaired sensation of rectal filling, and thereby potentially increasing the risk of incontinence (lack or perceived urge) and/or impaction (lack of perceived rectal filling). Our analysis of published data revealed somewhat inconsistent results which most likely reflect the wide spectrum of disease severity and often patchy distribution of sensory-motor losses in MS patients enrolled in these studies. Lastly, whole gut transit studies showed a slowed colon transit in MS patients with constipation. As normal colon transit times were seen in a disease control group with MS and fecal incontinence, this finding can clearly not be attributed to MS per se, but is rather a surrogate marker of constipation. The mere fact that colonic transit is slowed does not enable us to reliably distinguish the potential underlying mechanisms that might range from a true colonic problem (i.e., colonic inertia) to a secondary effect one would expect with impaired evacuation (i.e., dyssynergic defecation). These mechanistic studies largely focused on anorectal physiology. However, normal defecation behavior includes additional tasks that further confound the picture. Patients need to be able to reach toilet facilities, to be able to undress and transfer onto the toilet seat, and to initiate fecal expulsion with some increase in intra-abdominal pressure (straining). Limited mobility may also secondarily affect colonic function, further increasing the risk of constipation. Some or all of these issues are common in patients with moderate-to-advanced disability and have to be taken into account when we consider treatment options. 

Most of the reports on treatment of anorectal dysfunction in MS targeted some of the mechanisms described above. Several uncontrolled trials and case series employed behavioral interventions, augmented by biofeedback, to alter sphincteric function. While the interventions improved symptoms, these improvements were mostly limited to patients with mild disease. These observations are consistent with previously published results in patients without neuromuscular diseases, which showed good results in uncontrolled case series, but did not demonstrate superiority when patients with fecal incontinence were randomly assigned to control interventions or biofeedback [44–47]. From a conceptual standpoint, biofeedback for anorectal disorders requires intact neuromuscular control mechanisms to intentionally and effectively alter established, but undesired motor patterns. While compensation of limited losses may be possible under some circumstances, the approach does therefore not seem promising in MS patients with more significant disability. Published data appear to reinforce these theoretical concerns, which unfortunately limit the success of treatment for those who are truly in need of therapy. The situation is further confounded by coexisting sensory impairment and the impact of advanced disability with limited mobility. For example, aggressive laxative use is much more likely to result in soiling or even frank fecal incontinence, if sensory mechanisms do not provide a sufficiently early and/or intense sensation of urgency, or if patients cannot easily transfer onto a toilet seat. 

Nonspecific interventions, such as abdominal massage, may help, but do require significant effort without providing significant enough an improvement to truly offer a solution to patients with moderate or even more severe constipation. Milder problems will likely respond to many interventions, ranging from dietary to educational or medicinal approaches. In such situations, treatment should be simple, require limited time investment, integrate patient preference, and consider available resources. For patients with constipation, the typical recommendations advise that changes in fiber intake and activity should be considered as a first step [48, 49]. If these simple initial steps do not suffice, patients should then be instructed to the use of safe laxatives that allow flexible dosing, such as osmotically active agents (e.g., polyethylene glycol). If patients primarily complain about fecal incontinence, practitioners should first assess the influence of stool consistency on symptoms. Prior investigations have demonstrated that looser stools are the most important risk factor associated with fecal incontinence [50]. Consistent with these results, the use of loperamide for fecal incontinence was more effective than education, biofeedback, or diazepam in a randomized controlled trial [44]. 

While these simple steps may control problems in many patients, effective strategies are needed for individuals with persistent or more severe problems. Considering the often coexisting problems with impaired sensation and limited mobility, an ideal strategy for constipation should facilitate evacuation by softening bowel movements and perhaps even trigger colonic mass movements to help evacuation, without causing undue urgency at unanticipated or inopportune times. Cautious use of laxatives with appropriate instructions about flexible dosing remains important, but may need to be complemented by additional steps. Digital stimulation, a common practice in paraplegic individuals, may be adequate. The use of laxating suppositories may combine such a physical with a pharmacologic stimulus and thus be even more effective. In more severe forms of fecal incontinence, preemptive evacuation should be considered. Appropriately designed trials have clearly demonstrated that transanal irrigation effectively improves symptoms in patients with neurogenic forms of fecal incontinence [37, 51]. The approach is more labor- and time-intensive, making widespread acceptance more difficult. The use of laxating suppositories may first be tried as a simpler alternative and could be followed by cautious use of antidiarrheals, once evacuation has been accomplished. 

This survey of the published literature highlights the impact of anorectal dysfunction as a common manifestation of MS. Effective treatment is still largely missing for those who most need it. However, MS patients may be able to adapt simple strategies based on approaches used by patients with other neurogenic forms of bowel dysfunction. Conceptually, some of these strategies are analogous to the self-catheterization employed in bladder dysfunction. The relative simplicity, safety, and general availability support their use. However, these approaches need to be systematically studied to assess whether they are indeed effective. Testing these approaches in appropriately designed trials may also enable us to develop simple algorithms for anorectal dysfunction that can be used by patients, caregivers, and healthcare providers. The ultimate aim is to more effectively compensate for an impaired body function that is not as obvious as the inability to walk, is often not discussed, but still affects many MS patients on a daily basis, significantly contributes to the burden of caregivers, and has an often tremendous impact on the quality of life of patients and their family members.

## Figures and Tables

**Table 1:** Mechanisms of anorectal dysfunction.

Assessment tool	Symptom	Sample size	MS severity	Results	Reference
Anorectal manometry	Con	21	EDDS: 5.3	Decreased rectoanal inhibitory reflex	[23]
	Con or FI	39	EDSS: 5	No difference based on symptom patterns	[22]
			DSS ≤5: 5		
	Con	13	DSS ≥5: 8	Weak external sphincter: 62%; impaired straining: 82%	[11]
	Con	30	EDSS: 6	Lower squeeze pressure, impaired valsalva pressures	[12]
	Con and FI	11		Lower sphincter pressures in women	[35]
	Con and FI	23	Wheelchair: 9	Abnormal squeeze pressure in a subset	[36]
	Con and FI	16		Impaired amplitude and duration of squeeze pressure	[19]
	Con and FI	52	EDSS: 4.13	Decreased squeeze pressures	[16]
	FI	6		Markedly reduced squeeze pressure	[32]
	FI	12		Lower squeeze pressure in women only after childbirth	[27]
	Con	9	EDSS: 9.6	Decreased squeeze pressure	[13]

Recto-anal sensitivity	Con and FI	39	EDSS: 5	No differences in rectal or anal sensory thresholds	[22]
	Con and FI	11		Normal rectal sensory thresholds	[35]
	FI	5		Abnormal sensory threshold to distension in 3/5 patients	[19]
	Con	9	EDSS: 9.6	Normal rectal and anal sensory thresholds	[13]
	FI	6		Normal rectal sensory thresholds	[32]
	Con and FI	52	EDSS: 4.13	Normal rectal sensory thresholds	[16]
	Con and FI	30	EDSS: 6	Abnormal sensory threshold to distension in 15 patients	[12]

Con: constipation; FI: fecal incontinence; EDSS: expanded disability status scale.

**Table 2:** Treatment of anorectal dysfunction in multiple sclerosis.

Treatment	Sample size	Duration	Design	Results	Response rate	References
Biofeedback	39	3 months	Prospective series	Improvement of constipation and incontinence scores	46%	[22]
	13	4 months	Prospective series	Patient rating of biofeedback success positive: *n* = 5	38%	[11]
	18	2–6 months	Prospective series	Patient reported symptom improvement: *n* = 8	44%	[15]

Abdominal massage	30	8 weeks	RTC	Increase in defecation frequency for week 4 only; improved composite constipation scores weeks 4 and 8		[14]

Transanal irrigation	25	n/a	Retrospective series	Patient reported improvement: *n* = 10	40%	[37]
	10	n/a	Retrospective series	Patient reported improvement: *n* = 5	50%	[20]
